# How Standard of Truth Methodology Impacts Diagnostic PSMA-Targeting Radiopharmaceutical Evaluation: Learnings from the Phase 3 SPOTLIGHT Study

**DOI:** 10.3390/diagnostics15040473

**Published:** 2025-02-14

**Authors:** Benjamin H. Lowentritt, Albert Chau, Phillip Davis

**Affiliations:** 1 Chesapeake Urology Research Associates, Towson, MD 21204, USA; 2 Blue Earth Diagnostics Ltd., Oxford OX4 4GA, UK; albert@datacision.co.uk; 3 Blue Earth Diagnostics, Inc., Needham, MA 02094, USA; phillip.davis@blueearthdx.com

**Keywords:** histopathology, standard of truth, imaging, clinical trial, ^18^F-flotufolastat, diagnostic, PET, PSMA, recurrent prostate cancer

## Abstract

**Objectives**: To explore the impact of different standard of truth (SoT) methodologies on efficacy endpoints traditionally used in clinical trials of diagnostic radiopharmaceuticals, using data from the SPOTLIGHT study (NCT04186845) in patients with recurrent prostate cancer. **Methods:** Data from patients with baseline prostate-specific antigen (PSA) ≤ 5 ng/mL, who underwent ^18^F-flotufolastat imaging and had data for SoT determination, were reviewed. Majority-read patient level endpoints (verified detection rate [VDR] and patient-level positive predictive value [PPV]), and region-level PPV (in the prostate/prostate bed, pelvic lymph nodes, and extrapelvic sites) according to on-study reads by three blinded readers, were stratified by the SoT methodology (histopathology; post-PET confirmatory imaging; baseline/historic conventional imaging) used by the independent Truth Panel to verify ^18^F-flotufolastat-avid lesions. Differences between SoT groups for each endpoint were compared using a chi-square test (statistically significant if *p* < 0.0167). **Results**: Our analysis included 297 patients (median baseline PSA = 0.8 ng/mL): 56% (*n* = 166) had post-PET confirmatory imaging, 26% (*n* = 78) had baseline/historic conventional imaging, and 18% (*n* = 53) had histopathological confirmation of ≥1 PET-positive lesion. For all endpoints assessed, the highest majority-read values were achieved with histopathology SoT. For histopathology versus baseline/historic conventional imaging, VDR (77%) was 3.6-fold higher (*p* < 0.0001), patient-level PPV (79%) was 2.2-fold higher (*p* < 0.0001), and region-level PPV (50%) was 3.7-fold higher in the prostate/prostate bed (*p* = 0.009); smaller increases were seen in majority-read PPV in the pelvic lymph nodes (77%; 1.5-fold) and other sites (75%; 1.3-fold), but these were not of statistical significance. **Conclusions:** These data illustrate how SoT methods can substantially impact efficacy endpoints traditionally used in clinical trials of diagnostic radiopharmaceuticals. Notably lower endpoint values are achieved with imaging SoT than with histopathology.

## 1. Introduction

Positron emission tomography (PET) radiopharmaceuticals targeting prostate specific membrane antigen (PSMA) receptors on tumor cells can visualize smaller lesions than conventional imaging methods (such as magnetic resonance imaging [MRI] or computed tomography [CT]) [1,2,3], helping to facilitate prostate cancer diagnosis [4] and early detection of recurrent disease [5].

PET-positive results can be confidently verified with histopathology standard of truth (SoT) measures [6,7,8]. Verifying PET-positive findings from an investigational agent without histopathology data can be challenging, however, obtaining tissue samples may not always be possible in patients with recurrent disease where performing multiple biopsies could be impractical or unsafe [7,9]. In such patients, follow-up with conventional imaging is often used as an alternative SoT despite its potential for lower accuracy than the investigational agent [7,10]. Standardizing image interpretations within and, where possible, between clinical trials is important because the SoT methodology used to verify PET findings can substantially impact endpoint measures [11].

The diagnostic PSMA-targeting PET radiopharmaceutical ^18^F-flotufolastat (^18^F-rhPSMA-7.3) is currently approved for clinical use in the USA in patients with recurrent prostate cancer, following findings from the phase 3 SPOTLIGHT study (NCT04186845) [12]. SPOTLIGHT took place during the COVID-19 pandemic, which widely restricted patient visits to clinical practice [13,14,15]. One quarter of patients in the SPOTLIGHT efficacy analysis population (EAP) had no histopathology or post PET confirmatory imaging data for verification of PET-positive results, leaving investigators with only baseline/historic conventional imaging for SoT assessments [7]. Considering the recognized challenges associated with verifying PET-positive lesions of investigational PSMA-targeting radiopharmaceuticals, we conducted an analysis of data from a subset of patients in the SPOTLIGHT study to examine the potential impact of SoT methodology on efficacy endpoints traditionally used in clinical trials of diagnostic radiopharmaceuticals.

## 2. Materials and Methods

The methods of SPOTLIGHT have been published previously [7]. Briefly, SPOTLIGHT enrolled adult men with elevated prostate-specific antigen (PSA) levels (≥0.2 ng/mL) and suspected recurrent prostate cancer, who underwent PET 50–70 min after intravenous administration of 296 MBq ^18^F-flotufolastat.

For SoT assessment, the protocol required histopathology ≤60 days post-PET or confirmatory conventional imaging (MRI, CT, ^18^F-fluciclovine-PET, or ^18^F-sodium fluoride-PET per site standard of care) ≤90 days post-PET. Three blinded readers evaluated PET scans, and majority-read values represented agreement between ≥2 readers. SoT was established by histopathology when biopsy data were available; alternatively, a separate Truth Panel established SoT following review of conventional imaging data.

Our analysis included all patients from the SPOTLIGHT EAP who underwent ^18^F-flotufolastat imaging and had data for SoT determination, and with baseline PSA ≤ 5 ng/mL. Efficacy endpoint data were stratified by the SoT methodology used to verify PET-positive lesions.

Key endpoints traditionally used in diagnostic clinical trials were evaluated: patient-level verified detection rate (VDR) and patient-level positive predictive value (PPV); and region-level PPV in the prostate/prostate bed, pelvic lymph nodes, and other extrapelvic sites (lymph nodes, bones, and soft tissue/parenchyma).

Differences between SoT groups for each endpoint were compared using a chi-square test. *p*-values < 0.0167 were considered statistically significant; the threshold for statistical significance was adjusted from <0.05 to account equally for each of 3 pairwise comparisons (histopathology vs. baseline/historic imaging; histopathology vs. post-PET confirmatory imaging; post-PET confirmatory imaging vs. baseline/historic imaging) per endpoint.

## 3. Results

### 3.1. Baseline Characteristics

Of 420 patients enrolled in SPOTLIGHT, 391 underwent ^18^F-flotufolastat-PET, 389 had an evaluable scan, and 366 with data for SoT assessment were included in the EAP. Of patients in the SPOTLIGHT EAP, 297 had baseline PSA ≤ 5 ng/mL (median baseline PSA = 0.8 ng/mL [range = 0.0–5.0]) and were included in this study: 56% (*n* = 166) had post-PET confirmatory imaging, 26% (*n* = 78) had baseline/historic conventional imaging only, and 18% (*n* = 53) had histopathological confirmation of ≥1 PET-positive lesion. Patient baseline characteristics are presented in Table 1.

### 3.2. Impact of SoT Methodology on Efficacy Endpoints

The highest majority-read values were obtained with histopathology across all evaluated endpoints.

**Figure 1:**
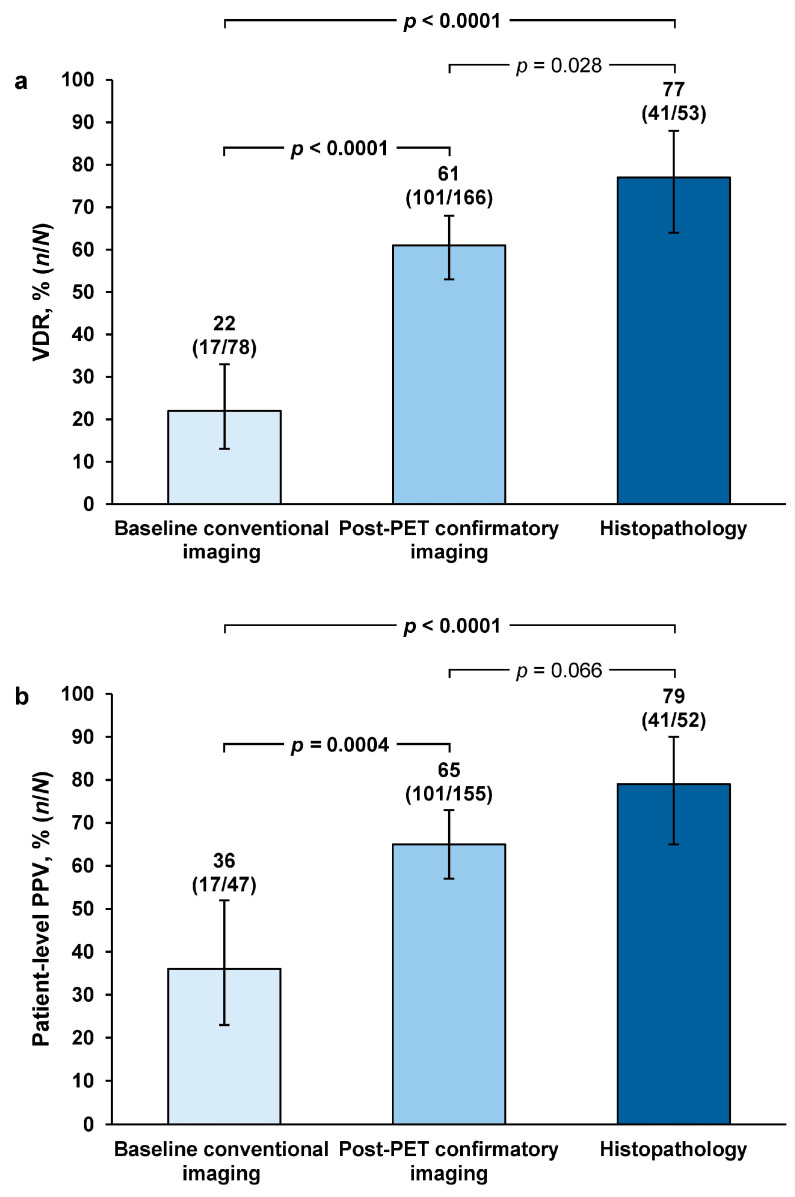
Majority-read patient-level (**a**) VDR and (**b**) PPV, stratified by SoT methodology. Error bars represent 95% confidence intervals. *p*-values adjusted for each of three pairwise comparisons (chi-square test); statistically significant *p*-values (<0.0167) are shown in bold font. PET: positron emission tomography, PPV: positive predictive value, VDR: verified detection rate.

Patient-level VDR was 77% (*n* = 41/53; 95% confidence interval [CI] = 64–88) among the 53 patients with histopathology data for SoT. This was 3.6-fold higher than the VDR estimate using baseline/historic conventional imaging as SoT. Using post-PET confirmatory imaging as SoT gave a 2.8-fold higher VDR estimate than baseline/historic conventional imaging. VDR estimates with histopathology and post-PET confirmatory imaging were significantly greater than VDR estimates with baseline/historic imaging (both *p* < 0.0001). Histopathology SoT gave a 1.3-fold higher VDR estimate than post-PET confirmatory imaging, but this difference was not of statistical significance (*p* = 0.028) (Figure 1a).

Patient-level PPV estimates with histopathology and post-PET confirmatory imaging were significantly greater than PPV estimates with baseline/historic conventional imaging: PPV was 2.2-fold higher with histopathology (*p* < 0.0001) and 1.8-fold higher when using post-PET confirmatory imaging (*p* = 0.0004) compared with baseline/historic conventional imaging. Histopathology SoT gave a 1.2-fold higher VDR estimate than post-PET confirmatory imaging, but this difference was not of statistical significance (*p* = 0.066) (Figure 1b).

**Figure 2:**
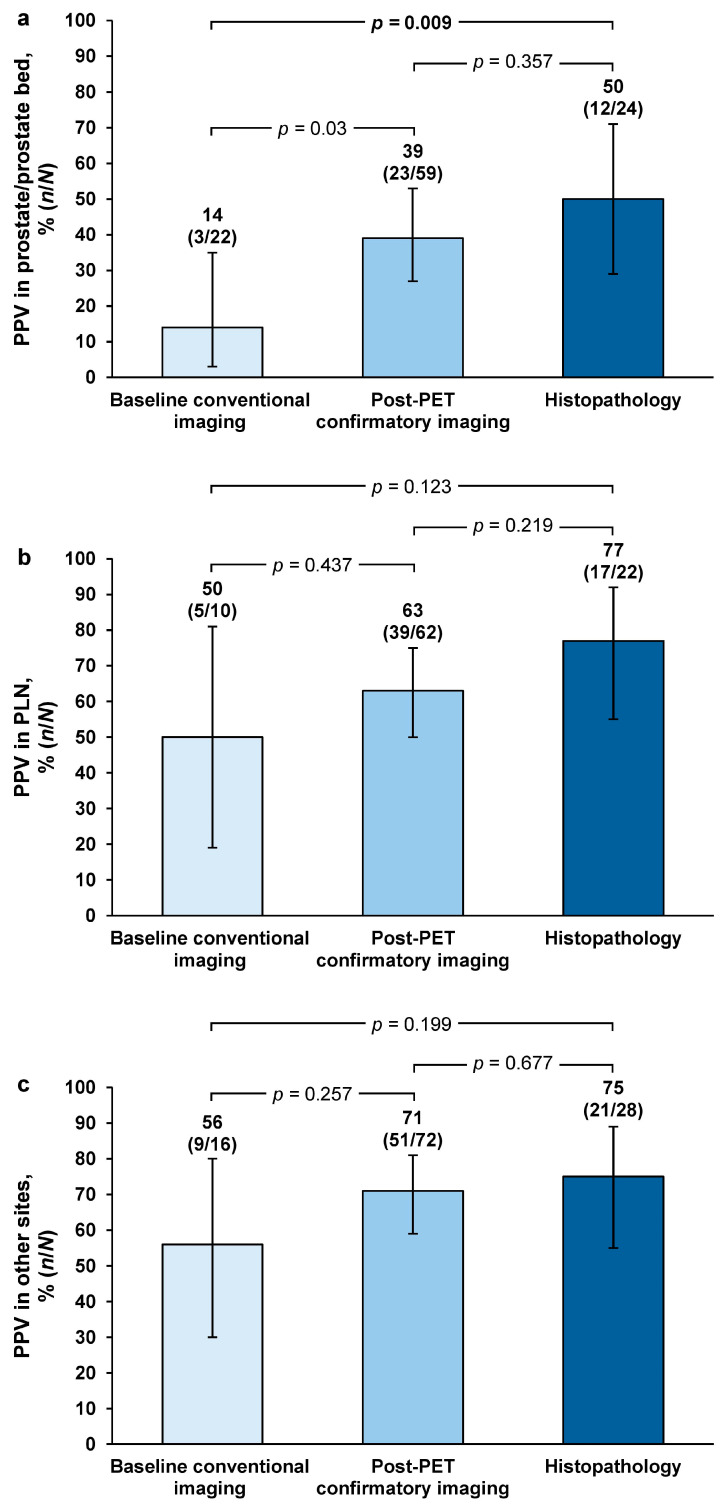
Majority-read region-level PPV in (**a**) the prostate/prostate bed, (**b**) pelvic lymph nodes, and (**c**) other extrapelvic regions, stratified by SoT methodology. Error bars represent 95% confidence intervals. *p*-values adjusted for each of 3 pairwise comparisons (chi-square test); statistically significant *p*-values (<0.0167) are shown in bold font. PET: positron emission tomography, PPV: positive predictive value.

For region-level PPV, majority-read values were highest with histopathology in the prostate/prostate bed (50%; *n* = 12/24 [95% CI = 29–71]) and pelvic lymph nodes (77%; *n* = 17/22 [95% CI = 55–92), and at other sites (75%; *n* = 21/28 [95% CI = 55–89]) (Figure 2).

Majority-read PPV for histopathology versus baseline/historic conventional imaging was 3.7-fold higher in the prostate/prostate bed, which was of statistical significance (*p* = 0.009). Majority-read PPV for histopathology versus baseline/historic imaging was 1.5-fold higher in the pelvic lymph nodes (*p* = 0.123) and 1.3-fold higher at other sites (*p* = 0.199). For post-PET confirmatory imaging versus baseline/historic imaging, majority-read PPV was 2.9-fold higher in the prostate/prostate bed (*p* = 0.03) and 1.3-fold higher in the pelvic lymph nodes (*p* = 0.437) and other regions (*p* = 0.257). For histopathology versus post-PET confirmatory imaging, majority-read values were 1.3-fold higher in the prostate/prostate bed (*p* = 0.357), 1.2-fold higher in the pelvic lymph nodes (*p* = 0.219), and 1.1-fold higher in other regions (*p* = 0.677) (Figure 2).

## 4. Discussion

SoT using histopathology consistently achieved the highest majority-read values for all evaluated endpoints. Post PET confirmatory imaging also provided increased majority-read estimates for all endpoints compared with baseline/historic conventional imaging. The SPOTLIGHT study noted that verification of PET-positive scans without histopathology could increase the rate of “false false positives”, and furthermore showed an increase in VDR and patient-level PPV in the per-protocol population, which excluded patients without histopathology or post-PET confirmatory imaging [7]. This highlighted the unsuitability of using only single-timepoint imaging for robust verification of PSMA-avid lesions, as also demonstrated by the results of our additional analysis.

While histopathology is the ideal SoT for prostate cancer diagnosis, challenges can arise where tissue biopsies are considered unfeasible or unsafe in patients with recurrent disease. Patients may not consent to undergo one or multiple tissue biopsies if they consider the procedure as uncomfortable or stressful [16], and undergoing multiple biopsies increases the risk of complications and infections [9]. Additionally, some PSMA-avid lesions may be too small to yield sufficient tissue samples for analysis. The nature of recurrent disease could have influenced the low proportion of patients with histopathology SoT data in SPOTLIGHT and, subsequently, in our analysis of SPOTLIGHT data (18%). It is also important to consider that patient visits to clinical practice at the time of the SPOTLIGHT study were limited by restrictions imposed during the COVID-19 pandemic [17,18]. Approximately 25% of patients in our analysis of SPOTLIGHT data did not have post-PET confirmatory imaging or histopathological follow-up, which was a likely consequence of these restrictions in patient visits. As our data show, reliance on only baseline or historic conventional imaging to verify imaging findings results in significantly lower endpoint estimates which ultimately would have impacted the overall study results. This is a clear limitation of SPOTLIGHT [7], given that such restrictions on patient visits to clinical practice did not affect earlier registration trials of PSMA-targeting radiopharmaceuticals [6,8].

Although post-PET confirmatory imaging could be considered a suitable alternative to histopathology when verifying an investigational PSMA-PET radiopharmaceutical, conventional imaging methodologies are notably less sensitive than PSMA-PET [1,2,3], and this was further supported by our data showing lower majority-read values for patient- and region-level endpoints with post-PET confirmatory imaging than with histopathology SoT. It should also be noted that use of ^18^F-fluciclovine-PET, which is more sensitive than CT in prostate cancer [19,20] and which was the most sensitive conventional imaging methodology available during SPOTLIGHT, was limited per US Food and Drug Administration request, and only 17% of lesions in SPOTLIGHT were verified with ^18^F-fluciclovine [7] (by contrast, 54% of patients in the ^18^F-DCFPyL registration trial had post-PET confirmatory imaging with ^18^F-fluciclovine [8]). Such a limitation on SPOTLIGHT SoT methodology, combined with varying rates of histopathology between SPOTLIGHT and other registration trials of PSMA-targeting radiopharmaceuticals [6,8], may have impacted performance estimates in the trials.

In our analysis of SPOTLIGHT data, statistical analysis of region-level data was limited by small sample sizes. Furthermore, slight differences in patient characteristics between groups were noted, including median baseline PSA values; although these differences between groups were minimal, any potential impact of these on the endpoint estimates would need to be determined by future prospective studies.

## 5. Conclusions

Our analysis of SPOTLIGHT data demonstrates the significant impact that SoT methods used to verify positive scan findings can have on clinical trial endpoints. These data highlight a limitation of evaluating endpoints with different SoT methodologies in diagnostic clinical trials; standardizing SoT measures in clinical trials could help improve the robustness of findings. Histopathology was the optimal SoT methodology that achieved significantly higher majority-read patient-level endpoint estimates than imaging SoT.

## Figures and Tables

**Table 1:** Baseline characteristics of patients from SPOTLIGHT with baseline PSA ≤ 5 ng/mL.

		SoT Methodology		
	Combined SoT (All Evaluable Patients)	Baseline/ Historic Conventional Imaging	Post-PET Confirmatory Imaging	Histo- Pathology
Patients, *N* (%)	297 (100)	78 (26)	166 (56)	53 (18)
Age, years				
*Median*	69	69	70	68
*Range*	43–85	43–85	45–85	50–83
Baseline PSA, ng/mL				
*Median*	0.8	0.49	0.94	1.4
*Range*	0.0–5.0	0.0–4.6	0.2–5.0	0.1–4.9
ISUP Grade Group, *n* (%)				
1	24 (8.1)	10 (13)	11 (6.6)	3 (5.7)
2	78 (26)	25 (32)	45 (27)	8 (15)
3	95 (32)	24 (31)	52 (31)	19 (36)
4	31 (10)	5 (6.4)	21 (13)	5 (9.4)
5	52 (18)	7 (9.0)	30 (18)	15 (28)
*Missing*	17 (5.7)	7 (9.0)	7 (4.2)	3 (5.7)
Prior radical prostatectomy, *n* (%) *	252 (85)	73 (94)	136 (82)	43 (81)
*With radiotherapy* ^†^	110 (44)	23 (32)	71 (52)	16 (37)
*Without radiotherapy* ^†^	142 (56)	50 (68)	65 (48)	27 (63)
Prior radiotherapy only, *n* (%) *	42 (14)	5 (6.4)	28 (17)	9 (17)

* Three patients who did not undergo radical prostatectomy or radiotherapy were excluded from column counts; ^†^ percentages calculated from number of patients with prior radical prostatectomy. ISUP: International Society of Urological Pathology, PSA: prostate-specific antigen, SoT: standard of truth.

## Data Availability

The original contributions presented in this study are included within the article itself. Further inquiries can be directed to the corresponding author.

## Abbreviations

The following abbreviations are used in this manuscript:

PET	Positron emission tomography
PSMA	Prostate-specific membrane antigen
MRI	Magnetic resonance imaging
CT	Computed tomography
SoT	Standard-of-truth
^18^F	Fluorine-18
EAP	Efficacy analysis population
PSA	Prostate-specific antigen
VDR	Verified detection rate
PPV	Positive predictive value
CI	Confidence interval
ISUP	International Society of Urological Pathology
